# Research on Apple Surface Disease Detection Method Based on Improved YOLOv11s

**DOI:** 10.3390/foods15091581

**Published:** 2026-05-04

**Authors:** Dongliang Liu, Yan Li, Xiaona Song, Luyang Feng, Jinxing Niu

**Affiliations:** 1 School of Mechanical Engineering, North China University of Water Resources and Electric Power, Zhengzhou 450045, China; 2 Shanghai Institute of Infectious Disease and Biosecurity, Fudan University, Shanghai 200433, China

**Keywords:** improved YOLOv11s, apple surface disease, GAM attention mechanism, defect detection

## Abstract

Apple surface diseases are crucial factors affecting the quality and yield of apples. Traditional manual inspection methods suffer from low efficiency and poor real-time performance. To address these issues, this paper proposes an apple surface disease detection method based on an improved YOLOv11s. Firstly, three groups of GAM attention mechanisms are integrated into the neck structure of the YOLOv11s to enhance the efficiency of feature fusion and the capability of semantic information transmission. Secondly, the original convolutional downsampling in the backbone network is replaced with a Haar-based feature downsampling module, enabling the model to retain more high-frequency detail information during the downsampling process. In addition, the WFU module is introduced to realize the dynamic allocation of feature weights, enhancing the model’s ability to recognize multi-scale defect features. Finally, the PIOUv2 loss function is adopted to optimize bounding box regression, improving the model’s detection performance for tiny defect spots. In addition, various data augmentation methods for small datasets are employed to improve the model training performance and effectively avoid the problem of data overfitting. The experimental results demonstrate that the F1-score of the proposed model is increased by 4.2%, and the mAP@50:95 is boosted by 2.4%. The detection performance outperforms various comparative models, which verifies the effectiveness and superiority of the proposed method.

## 1. Introduction

As a bulk fruit with extremely high economic value worldwide, apples are prone to surface defects caused by pest and disease infection, environmental stress and mechanical damage during the growth cycle. Among these influencing factors, pest and disease invasion is the primary inducement, which directly leads to severe yield loss and significantly reduces the fruit commercial grade and overall economic benefits of the apple industry chain [1,2]. Meanwhile, with the continuous expansion of apple cultivar diversity and the increasing market demand for high-quality fruits, pest and disease defects have also become a key constraint restricting the promotion of high-quality varieties and the enhancement of market competitiveness [3,4]. Therefore, realizing accurate and efficient detection of apple surface diseases is of vital practical significance for promoting the high-quality and sustainable development of the apple industry.

At present, the traditional manual visual inspection is still the mainstream detection method in most orchards. Although this method requires no complex equipment and is applicable to small-scale orchards, it has prominent and inherent limitations. It is not only time-consuming and labor-intensive, with low detection efficiency, but also difficult to adapt to the needs of large-scale orchards. To break through the bottlenecks of traditional manual detection and drive the transformation of apple pest and disease detection towards automation and precision, domestic and foreign scholars have carried out extensive in-depth research and proposed a variety of deep learning-based detection schemes for apple surface diseases. These studies cover core fields, such as disease identification, target location, and model optimization, laying a solid technical foundation for the precise prevention and control of apple diseases and pests. The core research progress can be summarized into three major directions.

The first direction is the research, development and optimization of specialized disease detection models. Assad et al. [5] constructed the AppleNet multi-class classification model based on the ResNet50 convolutional neural network and transfer learning strategy, which is dedicated to the rapid detection and accurate classification of various apple diseases, achieving a high classification accuracy of 96.00% on the dataset collected in actual orchard scenarios, effectively improving the accuracy and efficiency of disease detection. Wang et al. [6,7] proposed the CA-ENet and other improved deep learning models, which optimized the feature extraction process by embedding attention mechanisms to filter redundant feature information, showing strong anti-interference performance in apple leaf disease identification tasks and effectively improving the identification accuracy. Aggarwal et al. [8] put forward the MEFN framework, which innovatively integrates RGB, thermal imaging, and hyperspectral image data, solving the problem of the weak generalization ability of models based on a single data type, and its inference speed meets the deployment requirements of mobile devices and Internet of Things terminals. Srinivasan et al. [9] proposed a dual-branch attention-guided vision network (DBA-ViNet), which, combined with Grad-CAM visualization technology, not only improves the accuracy of fruit disease identification, including apples, but also enhances the interpretability and credibility of the model. Ashurov AY et al. [10] proposed a lightweight model that integrates depthwise separable convolution, an SE attention module, and improved residual skip connections. This model can be used for efficient and high-precision automatic plant disease identification, balancing performance and computational cost, and is suitable for practical agricultural deployment. Zhang et al. [11] built a multi-class dataset covering apple varieties, diseases, and freshness, and the Optimized Apple Orchard Model (OAOM), which can realize simultaneous variety identification, disease diagnosis, and freshness evaluation, with a disease identification accuracy of 99.66%. Ma B et al. [12] designed the HDC-Net, targeting the particularity of apple aphid detection, realizing the density grading detection of apple aphids, and providing a targeted solution for precise pest control.

The second direction is research on target localization and lightweight model design. Zhou et al. [13,14,15] improved the classical YOLO model to realize instance segmentation and 3D localization of multi-colored and different maturity apples, providing reliable technical support for the precise operation of orchard robotic arms. Wang et al. [16] proposed the LPNet lightweight progressive network, which can accurately and rapidly estimate apple fruit posture, and its lightweight structure matches the limited computing resources of orchard robots. Olguín-Rojas et al. [17] developed a lightweight YOLO architecture and successfully deployed it on embedded systems, solving the hardware adaptation problem of on-site orchard detection. Geng et al. [18] combined HSV color space transformation with the YOLOv8 model, enhancing the accuracy of apple identification, counting, and maturity evaluation in complex orchard environments, and reducing the interference of lighting changes and branch occlusion. Yuan et al. [19] put forward the improved RFE-YOLO model, which introduced an efficient feature pyramid and attention module to further boost the detection precision of apple fruit defects, supporting the rapid screening of defective fruits.

The third direction is the improvement of auxiliary technical support systems. Seilov et al. [20] proposed a multi-modal data fusion detection method, focusing on optimizing the preprocessing process of outdoor depth images and improving the anti-noise performance of apple detection in dense planting orchards. Gao et al. [21] developed an integrated apple defect detection and quality grading system and introduced the Jump Loss function to address the class imbalance problem in detection, significantly optimizing the overall model performance. Wang Z et al. [22,23] integrated instance segmentation, edge perception, attention mechanism, and natural language processing, providing comprehensive decision support for apple quality detection and growth abnormality prediction. Wang D et al. [24] constructed a dedicated dataset for small apple fruits, supplying reliable data support for target detection research in the early fruit thinning stage. Hu X [25] solved the low recognition rate of apple surface detection, including rot, disease, tear, and mechanical damage under small sample conditions, and realized automatic fruit grading. Liu J et al. [26] conducted research on the online detection of apple maturity and fruit diameter, promoting the progress of visual technology for intelligent picking and facilitating the intelligent upgrading of orchards.

Although the above deep learning-based studies have achieved remarkable progress in apple disease and defect detection, there are still core shortcomings that limit their practical application in large-scale apple detection scenarios. Most existing models suffer from weak generalization ability, high computational complexity, and insufficient detection accuracy of diseases and tiny defects; some models lack interpretability, and have insufficient matching between dataset support and lightweight design, making it difficult to deploy on portable and embedded edge devices for field application. To tackle the above deficiencies and meet the urgent demand for lightweight, rapid, and precise disease and defect detection technology in large-scale apple production, this paper focuses on the accurate identification of apple surface diseases and defects. Taking the YOLOv11s model as the baseline, a series of targeted structural improvements and algorithm optimizations are conducted to construct a lightweight and high-efficiency detection model. The proposed model aims to realize the rapid and accurate identification of various pests, diseases, and tiny defects on apple surfaces, and is compatible with the deployment of portable and embedded detection equipment, so as to provide reliable technical support for precise apple quality detection and boost the high-quality development of the apple industry.

## 2. Experimental Dataset

This paper adopts a hybrid dataset, combining a public dataset [27] and a self-built private dataset. The dataset contains four categories of apple images: 7759 healthy apple images, 281 apple blotch images, 310 apple rot images, and 349 apple scab images, totaling 8699 original annotated samples.

Given that the ratio of healthy to diseased samples in the dataset is 8.25:1, the significant class imbalance may lead to model overfitting and distorted evaluation metrics [28,29]. To mitigate the adverse impact of class imbalance on model performance, data augmentation is applied to the diseased samples in this study. Methods including rotation, Gamma correction, Contrast Limited Adaptive Histogram Equalization (CLAHE), and random occlusion are employed for sample expansion. Furthermore, to prevent data leakage, the augmentation is performed strictly after dataset splitting.

The dataset splitting and augmentation procedure is as follows: First, 200 healthy apple images, 65 apple blotch images, 75 apple rot images, and 85 apple scab images are randomly selected to form the validation set. The test set is constructed using the same sampling strategy and proportion. Subsequently, all remaining diseased samples are randomly rotated by either 90° or 180°. After rotation, both the original diseased samples and the newly generated rotated samples are randomly processed using either Gamma correction or CLAHE. The Gamma correction parameter is randomly selected within the range of 0.8 to 1.3, and the CLAHE mask size is set to 12 × 12. Finally, half of the processed diseased samples are randomly selected to undergo random occlusion, completing the data augmentation process.

**Figure 1:**
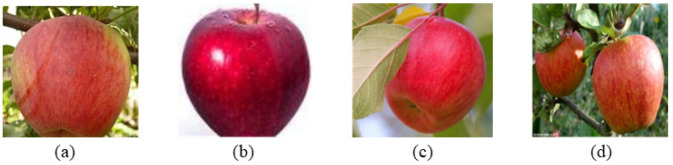
Healthy apples (**a**–**d**).

**Figure 2:**
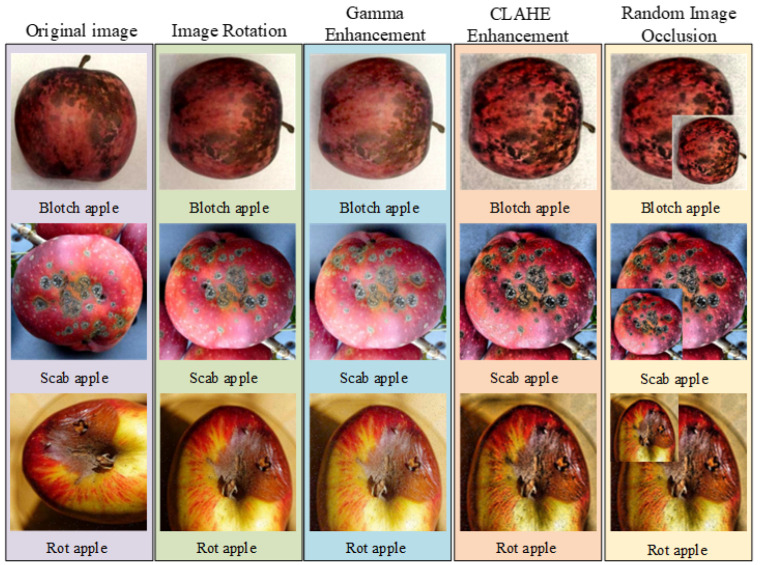
Diseased Apple Images and augmentation results.

Through the aforementioned strategy, the remaining diseased samples are expanded to six times their initial quantity. After expansion, the training set contains 7359 healthy apple images and 2940 diseased apple images. The diseased category specifically comprises 906 apple blotch images, 960 apple rot images, and 1074 apple scab images. Examples of the augmented images for healthy and diseased apples are shown in Figure 1 and Figure 2, respectively.

## 3. Enhanced Model Architecture

### 3.1. YOLOv11

As a classic one-stage object detection model, YOLO (You Only Look Once) is mainly composed of three modules: the backbone network, neck layer and detection head. Benefiting from the end-to-end architecture of one-stage detection, it performs significantly better than traditional two-stage detection models in both detection speed and accuracy. As the latest iterative version of the YOLO series, YOLOv11 inherits the core one-stage detection philosophy of “You Only Look Once”. By simplifying the backbone network structure and optimizing the design of feature fusion modules and loss functions, this paper adopts YOLOv11 as the baseline model, which effectively improves small object detection accuracy and robustness in complex scenarios while ensuring real-time inference efficiency (FPS). The backbone of YOLOv11 inherits the convolution, residual connection and SPPF (Spatial Pyramid Pooling-Fast) structures of the YOLO series to extract multi-scale features. It introduces the C3K2 module to dynamically adapt to different object scales, and embeds a multi-head position-sensitive attention mechanism at the end of the backbone to enhance feature representation of key regions. The neck layer adopts the PAFPN (Path Aggregation Feature Pyramid Network) architecture and realizes the complementary enhancement of shallow detail features and deep semantic features through bidirectional fusion from top to bottom and bottom to top.

**Figure 3:**
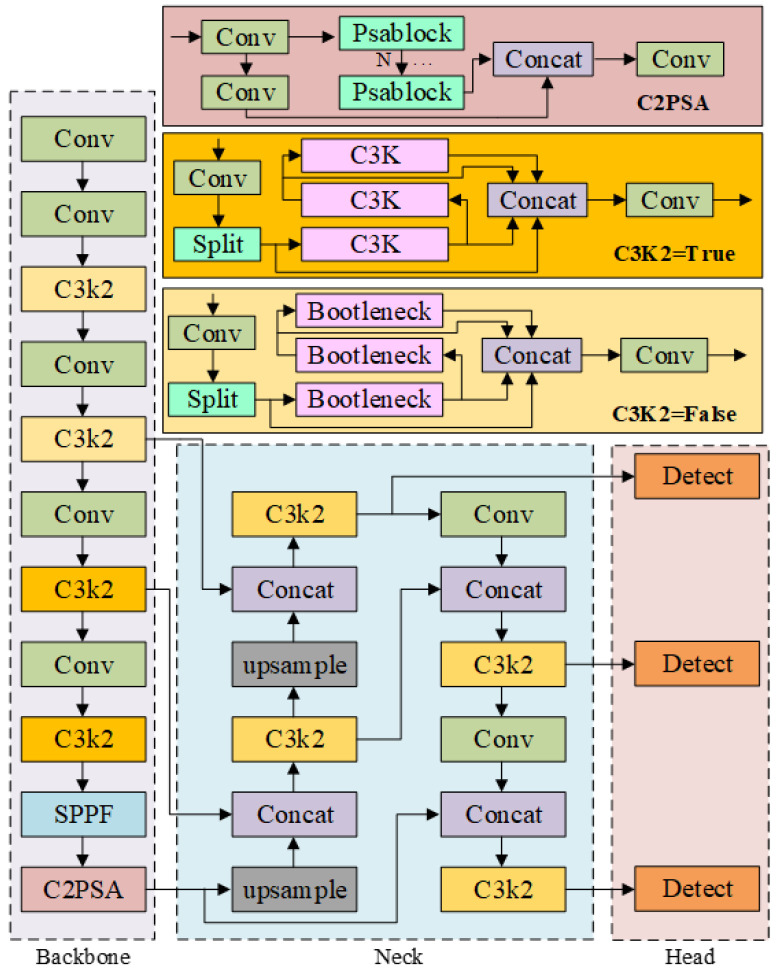
The network structure of the YOLOv11.

Compared with previous models in the YOLO series, YOLOv11 significantly improves detection accuracy and speed through structural optimization, achieving a balance between accuracy and efficiency. The network structure of the YOLOv11 model is shown in Figure 3 below.

### 3.2. GAM Attention Module

The neck structure of YOLOv11 can effectively achieve feature fusion and semantic information transmission. However, in lightweight versions of the YOLOv11 model, such as YOLOv11s and YOLOv11n, the channel dimension of feature maps is relatively small, resulting in insufficient spatial information representation capability. This limitation lowers the efficiency of information transmission from the bottom layer to the top layer, causing part of the feature information to be lost during the transmission process. In addition, the lesions on the apple surface are generally characterized by small-scale, abstract, and fuzzy features and complex backgrounds, which are extremely prone to missed detection and false detection during the detection process.

**Figure 4:**
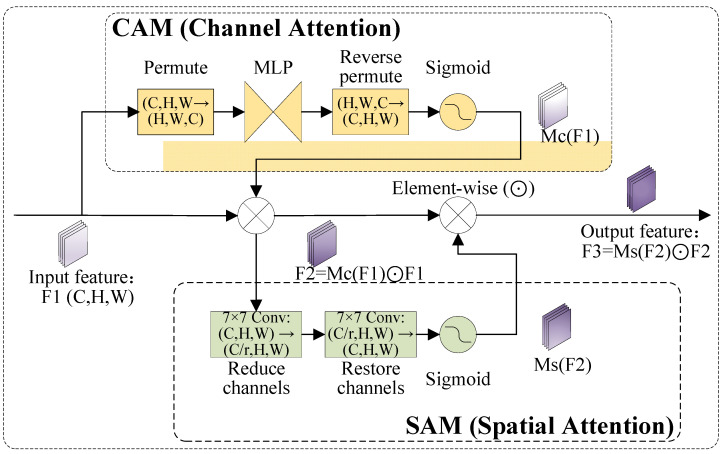
Structure diagram of GAM attention mechanism.

To address the above problems, this study improves the YOLOv11s model by embedding the Global Attention Mechanism (GAM) into the neck module, aiming to enhance the model’s performance in identifying and classifying diseases on apple surfaces. The GAM attention mechanism is improved based on the Convolutional Block Attention Module (CBAM), adopting a serial structure of “channel attention–spatial attention” and realizing cross-dimensional (channel–spatial) attention modeling through two functionally complementary sub-modules. In the channel attention sub-module, the 3D permutation strategy is adopted to retain the three-dimensional information of the feature map, namely channel, height, and width, effectively avoiding the loss of spatial details caused by the traditional global pooling operation. Then, two fully connected layers (MLPs) are used to amplify the cross-dimensional dependency between the channel and spatial dimensions, and generate adaptive channel attention weights. In the spatial attention sub-module, double convolutional layers are first used to replace the traditional pooling operation, and spatial context information is directly extracted through convolution calculation to avoid information compression loss during pooling. Then, group convolution is introduced to restrain the excessive growth of parameters, and the channel shuffle mechanism is combined to ensure cross-channel feature interaction. The entire spatial attention module adopts a non-pooling design, retaining the original size of the feature map throughout the process to further improve spatial positioning accuracy. The channel attention and spatial attention adopt a serial cascade architecture. For the input feature map with the size of F_1_ ∈ *R^C^*^×*H*×*W*^ (where *C* represents the number of channels, and *H* and *W* represent the height and width of the feature map, respectively), the weighted feature map F2 is output after channel attention processing, and the final weighted feature map F3 is obtained after optimization by the spatial attention module. The core process is shown in Formulas (1) and (2). The specific structure of the GAM attention mechanism is shown in Figure 4. Herein, *M_C_* denotes the channel attention map, *M_S_* denotes the spatial attention map, and ⊗ represents Element-wise multiplication.

(1) $$F_{2}=M_{C}(F_{1})⊗F_{1}$$

(2) $$F_{3}=M_{S}(F_{2})⊗F_{2}$$

### 3.3. Haar Wavelet Downsampling

When the YOLOv11s model performs multi-scale feature fusion, it is necessary to conduct dimensionality reduction and downsampling on feature maps of different sizes to achieve size matching. Although the traditional convolutional dimensionality reduction and downsampling method can compress feature dimensions and reduce computational cost, it can lead to the loss of high-frequency detailed information and impair the detection performance of tiny targets and fine-grained features.

To retain high-frequency details, this paper adopts the Haar wavelet transform to replace traditional convolutional downsampling, achieving more robust multi-scale feature extraction without increasing the number of parameters or computational complexity. This approach effectively preserves fine details, such as lesion edges and tiny textures, and significantly enhances the model’s ability to discriminate subtle features, and improves overall detection performance.

The Haar wavelet transform can decompose a discrete signal *x* = [*X*_0_, *X*_1_, *X*_2_ … *X_N_*], with length *N*, into a low-frequency approximate component and a high-frequency detail component: the coefficients of the low-frequency approximate component are obtained by averaging the adjacent signal sampling points, which are used to retain the overall trend of the signal; the coefficients of the high-frequency detail component are obtained by differencing the adjacent signal sampling points, which are used to capture the edges and local details of the signal. The calculation formula is shown in Formula (3).

(3) $$\left\{\begin{array}{l} \alpha_{k1}=\frac{x_{2k}+x_{2k+1}}{\sqrt{2}}\\ \alpha_{k2}=\frac{x_{2k}-x_{2k+1}}{\sqrt{2}} \end{array}\right.$$

Herein, *α_k_*_1_ denotes the low-frequency approximate component, *α_k_*_2_ denotes the high-frequency detail component, *x*_2*k*_ represents the value of the 2*k*-th sampling point in the discrete signal, and *x*_2*k*+1_ represents the value of the (2*k* + 1)-th sampling point immediately following *x*_2*k*_ in the discrete signal.

**Figure 5:**
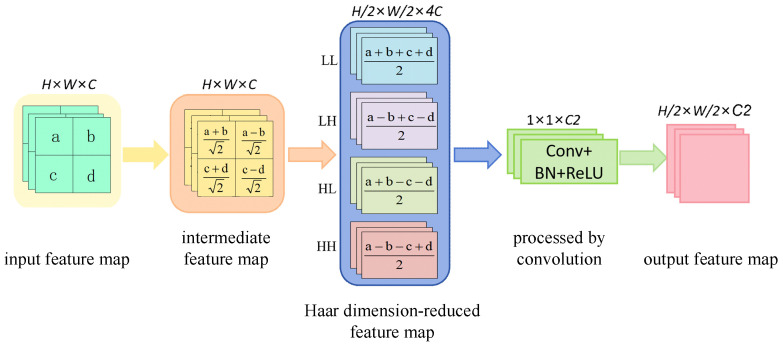
Schematic diagram of Haar dimension-reduction process for 2D image features.

For two-dimensional image features, downsampling based on the Haar wavelet transform can be performed sequentially in the horizontal (row) and vertical (column) directions, and finally, four types of feature components are output. The specific process is as follows: first, perform a one-dimensional Haar wavelet transform row by row on the input feature map with a size of *H* × *W* × *C* to obtain an intermediate feature map with the same size of *H* × *W* × *C*, which contains low-frequency and high-frequency components in the row direction. Then, perform a one-dimensional Haar wavelet transform column by column on the intermediate feature map after row transformation to obtain a wavelet-transformed feature map with a size of *H*/2 × *W*/2 × 4*C*, which consists of a low-frequency approximate feature (LL), a horizontal detail feature (LH), a vertical detail feature (HL) and a diagonal detail feature (HH). Finally, *C*2 1 × 1 convolutions are used to perform weighted fusion on the above four groups of features, followed by normalization and activation function processing, in sequence, to obtain the final dimension-reduced output feature map. The detailed process of this feature transformation is shown in Figure 5. This study replaces the two convolutional downsampling modules in the neck structure of the YOLOv11s with Haar wavelet downsampling, which not only reduces the computational cost of the model but also effectively improves the model’s ability to detect tiny targets and detailed features.

### 3.4. Weighted Fusion Unit Module

In the YOLO series of multi-scale object detection models, high-resolution shallow features contain rich detailed information and are suitable for small object detection tasks; low-resolution deep features have stronger semantic information expression ability and are more suitable for large object detection tasks. Most traditional feature fusion methods adopt simple concatenation or feature addition, assigning equal weights to features of different scales, and fail to fully consider the differentiated contributions of features at each scale in specific detection tasks.

To further improve the detection accuracy and effect of tiny lesions on apple surfaces, this paper embeds the Weighted Fusion Unit (WFU) into the YOLOv11s model. This weighted fusion unit can assign dynamic weights according to the importance of features from different sources, enabling the model to automatically focus on more valuable feature information based on the actual needs of the current tiny apple surface lesion detection task, effectively suppress the interference of invalid features, and, thus, significantly improve the detection accuracy of the model. The mathematical expression of the weighted fusion unit is shown in Formula (4). Where Fout represents the output fused feature map; *w_i_* represents the number of input features; *T_i_* denotes the dynamic weight of the *i*-th feature; and *F_i_* stands for the feature transformation function and refers to the *i*-th input feature map.

(4) $$F_{\mathrm{out}}=\sum_{i=1}^{n}w_{i}\cdot {Τ}_{i}(F_{i})$$

**Figure 6:**
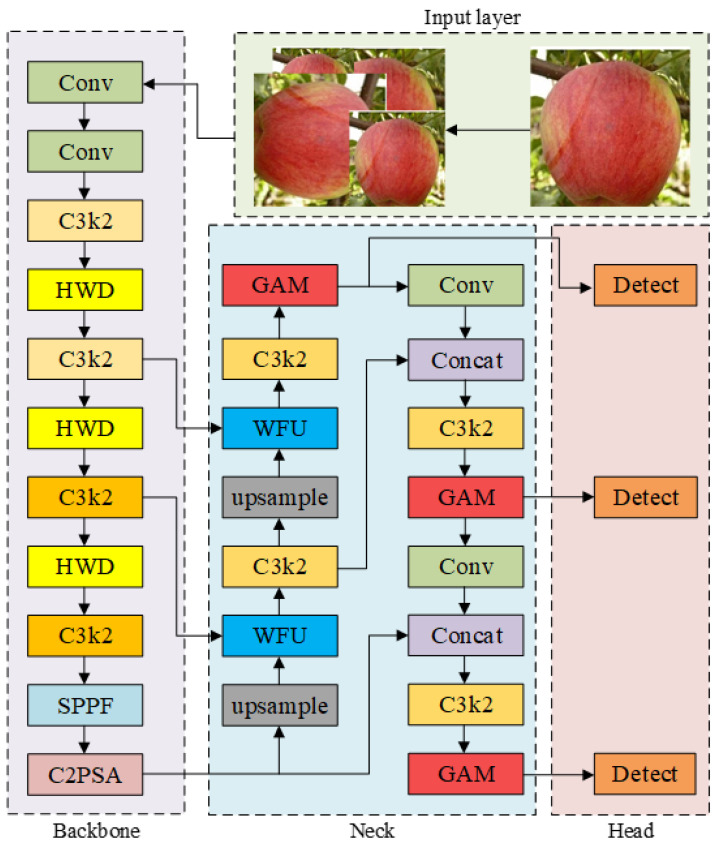
The structure of the improved YOLOv11s model.

In this paper, three groups of WFU modules are added to the backbone network of the YOLOv11s, enabling the model to automatically assign feature weights according to feature characteristics, so as to improve the detection capability of the model. The structure of the final improved model is shown in Figure 6.

### 3.5. Improved Loss Function

The YOLOv11s model adopts the CIOU loss function as the localization loss function. Although it introduces penalty terms for the center distance and aspect ratio consistency of bounding boxes on the basis of traditional IoU, and solves the problem that IoU is insensitive to the positioning deviation of bounding boxes to a certain extent, CIOU has obvious limitations when facing small target detection scenarios, such as disease spots and tiny defects on apple surfaces, as it is sensitive to the scale change of small-scale targets and has weak shape fitting ability, which is prone to false detection and missed detection.

To address the above problems and improve the localization performance of YOLOv11s in detecting tiny disease spots on apple surfaces, this paper introduces the PIoUv2 loss function into the localization loss calculation of YOLOv11s. As an improved version of PIoU, PIoUv2 accurately captures the boundary detail offset of small targets through the normalized boundary position deviation term P, making up for the defect that CIOU only focuses on the center point. At the same time, it adopts a double-layer exponential smoothing penalty function and introduces an adjustable coefficient *λ*, which not only strengthens the nonlinear penalty for small target position deviation but also avoids loss oscillation during the training process. In addition, PIoUv2 normalizes the position deviation by the size of the ground-truth box, eliminating the problem of unbalanced penalty caused by the scale difference of small targets, and can adapt to the localization requirements of disease spots of different sizes, effectively improving the localization accuracy and detection stability of YOLOv11s for small targets. The PIoUv2 loss function is shown in Formula (5). Where *λ* is the hyperparameter, which is set to 1.3 in this study; *p* represents the penalty term related to target size; and IOU denotes the intersection over union between the ground-truth box and the predicted box.

(5) $$\left\{\begin{array}{l} L_{piouv1}=1-IOU+e^{-p^{2}}\\ L_{piouv2}=3(\lambda p)e^{-(\lambda p)2}\cdot L_{piouv1} \end{array}\right.$$

## 4. Experiment and Analysis

### 4.1. Experimental Environment

The experiment was completed on a high-performance workstation. The workstation is equipped with an Intel i9-14900K processor, an NVIDIA GeForce RTX 5090 Graphics Processing Unit (GPU), 64 GB DDR5 RAM, and runs the Windows 11 Professional operating system. The model adopted in the experiment was implemented based on Python 3.11.9, and the PyTorch-1.13.1 and cuda126 deep learning frameworks were used to complete the training and inference processes of the model.

The main training hyperparameters and model preprocessing hyperparameters used in the experiment are shown in Table 1.

### 4.2. Dataset Augmentation Effect Experiment

To validate the rationale behind the dataset balancing operation, this study compares the performance of the YOLOv11s and YOLOv10s models before and after dataset augmentation. The original dataset underwent no data augmentation processing, and its test and validation sets remain identical to those of the augmented dataset. The model training process employed the same hyperparameters and was conducted over multiple training runs. The experimental results presented are the optimal values obtained from these multiple training sessions, as shown in Table 2 and Table 3.

The experimental results demonstrate that after dataset augmentation, the models exhibit significant improvement in the recognition performance of diseased apples. Specifically, after augmentation, the prediction accuracy for blotch apples increased by 8.1% and 6.3% on the YOLOv10s and YOLOv11s models; for rot apples, the prediction accuracy increased by 6.2% and 7.3%; and for scab apples, the prediction accuracy increased by 2.3% and 3.5%, respectively. Meanwhile, All Class mAP@0.5 improved by 4.8% and 4.9% on the YOLOv10s and YOLOv11s models, respectively. Dataset augmentation enables the models to learn the features of diseased apples more comprehensively, thereby significantly enhancing detection performance, and thus validates the rationale of the adopted augmentation methods.

### 4.3. Ablation Experiment

To verify the rationality and advancement of the model structure improvement method, ablation experiments were carried out in this study, which compared the performance of the model in apple disease detection tasks before and after the improvement.

Five models were designed in the ablation experiments to compare and verify the improvement effect, which are detailed as follows: Model 1 is the basic YOLOv11s model structure with the CIoU loss function; Model 2 adds three groups of GAM attention modules on the basis of Model 1; Model 3 replaces the original Conv module with a Haar-based feature downsampling module on the structure of Model 2; Model 4 further adds a feature fusion module based on the Weighted Fusion Unit (WFU) on the basis of Model 3; and Model 5 replaces the loss function with the PIoUv2 loss function on the basis of Model 4.

**Figure 7:**
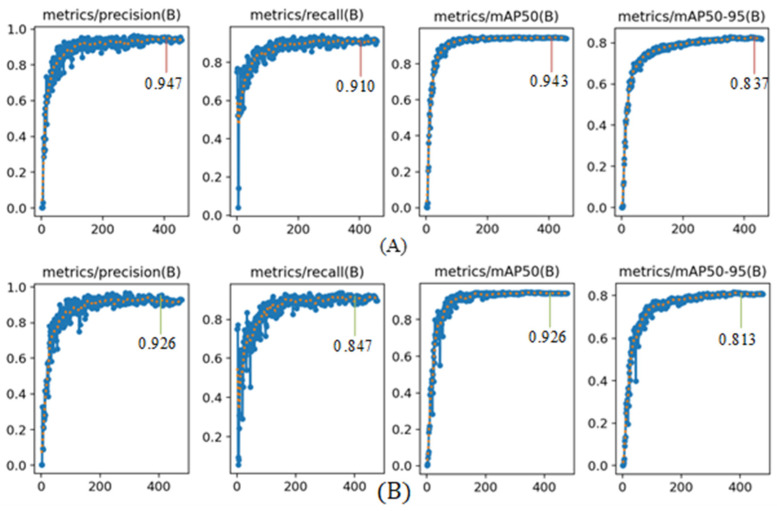
Comparison chart of performance metrics before and after model improvement.

The above five groups of models were trained with the hyperparameters in Table 1; the number of training epochs was set to 1000, and the early stop patience was set to 100. The early stopping strategy means that if the mAP@50:95 index of the model does not improve in 100 consecutive training epochs, the training will be terminated early. The model training is based on the balanced dataset, and multiple rounds of training and testing were carried out for all five groups of models. The optimal model performance is shown in Table 4. The comparison of detection performance indicators before and after model improvement is shown in Figure 7, where (A) is the improved model and (B) is the baseline model.

According to the comparison results of the ablation experiments in Table 4, it can be seen that the YOLOv11s base model achieved an F1-score of 0.886, mAP@50 of 0.926, and mAP@50:95 of 0.813 on the balanced apple disease detection dataset. After introducing three groups of GAM attention mechanisms into the neck structure of the YOLOv11s model, the F1-score, mAP@50, and mAP@50:95 of the model increased by 0.8%, 0.4%, and 0.5%, respectively. On this basis, the Haar feature downsampling module and HWF feature adaptive module were further introduced, and the F1-score, mAP@50, and mAP@50:95 of the model reached 0.917, 0.939, and 0.831, respectively, which significantly improved the ability to identify tiny disease defects. Finally, after replacing the loss function with PIoU, the F1-score, mAP@50, and mAP@50:95 of the model increased to 0.928, 0.943, and 0.837, respectively. Compared with the YOLOv11s base model, the three indicators increased by 4.2%, 1.7%, and 2.4% respectively.It can also be seen from Figure 7 that the improved model is significantly superior to the baseline model in four core indicators during training: precision, recall, mAP@50, and mAP@50:95. Furthermore, by comparing the mean and variance of the results from multiple training sessions, it can be observed that the model exhibits stable performance across repeated runs, with only minor deviations in the outcomes.

According to the experimental data in Table 5, all models can effectively identify healthy apples and three types of diseased apples, namely blotch apples, rot apples, and scab apples, and the overall detection performance is gradually improved with the optimization of the model structure. After the model improvement, the full-category mAP@50:95 increased from 0.813 of YOLOv11s to 0.837, with an increase of 2.4%; at the same time, the mAP@50 also achieved an increase of 1.7%. In the diseased apple recognition task, the recognition accuracy of the improved model for blotch apples reached 0.977, an increase of 2.7% compared with that before improvement. Although the recognition accuracy for rot apples was relatively low, it also increased from 0.888 before improvement to 0.922. Finally, the recognition accuracy for scab apples also increased by 1.4%. It can be concluded that the improvement of the model structure in this paper is reasonable and advanced, and is applicable to the apple disease detection task.

### 4.4. Comparison Experiment with Other Algorithms

To verify the effectiveness and superiority of the improved model, comparative experiments were conducted in this paper between the proposed model and several mainstream object detection algorithms. All models were trained with identical hyperparameters, and the experimental results are presented in Table 6.

As can be seen from Table 6, the improved YOLOv11s model outperforms other two-stage object detection algorithms in the task of apple surface disease and defect detection. On the premise of reducing the number of parameters and computational cost, the F1 score, mAP@50, and mAP@50:95 of the improved model are increased by 4.2%, 1.7%, and 2.4%, respectively, compared with the original model, while the detection speed remains basically unchanged. Parameters and higher computational cost, as well as two-stage detection models like Faster R-CNN, the improved model still exhibits significant advantages in both detection accuracy and speed.

**Figure 8:**
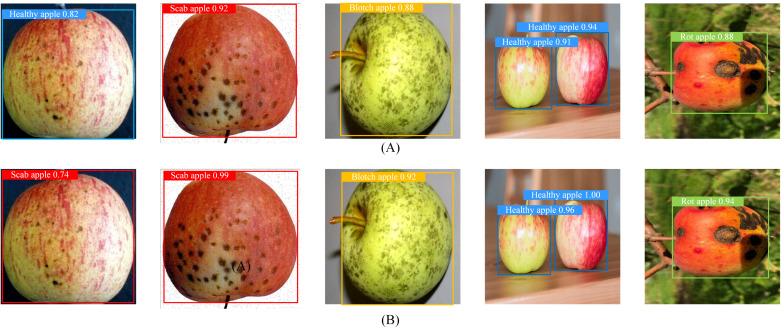
Apple disease prediction results.

The detection speed of the improved model can reach 556 FPS. Although this value is slightly lower than that of lightweight YOLOv10s series models, it still meets the industrial requirements for real-time detection of apple diseases. Finally, apple disease images were predicted using the original and improved models, respectively, and the results were displayed in Figure 8, where Panel (A) denotes the results of the original model and Panel (B) denotes those of the improved model. It can be seen from the detection results that compared with the unimproved YOLOv11s model, the proposed model delivers higher detection confidence and exhibits stronger capability in the recognition and localization of subtle defects.

## 5. Conclusions

Apple surface disease detection faces practical challenges, such as complex backgrounds, diverse and abstract defect morphologies, and weak features of tiny lesion spots. Traditional detection models have difficulty balancing accuracy and efficiency, and exhibit poor robustness in identifying tiny defects. To address these pain points, this paper proposes an apple surface disease detection method based on the improved YOLOv11s, upgrading the model performance through multi-dimensional optimizations: multiple groups of GAM attention mechanisms are embedded into the network structure to enhance the model’s perception and extraction capability for tiny disease features; the Haar-based feature downsampling module is adopted to replace traditional convolutional downsampling, retaining high-frequency detail information to the greatest extent; the WFU weighted fusion unit is introduced to realize dynamic allocation of multi-scale feature weights, focusing on effective features and suppressing redundant interference; and, the PIoUv2 loss function is selected to optimize the bounding box regression process, making up for the positioning defects of traditional CIoU loss in small target detection and improving the model’s positioning accuracy for tiny lesion spots.

The rationality and advancement of the proposed improved model have been verified through ablation experiments and multi-algorithm comparative experiments, and the model achieves an efficient balance between detection accuracy and inference speed. The experimental data show that the improved model achieves an F1-score of 92.8% and a detection speed of 551 FPS, with comprehensive detection performance superior to many mainstream object detection algorithms. It can accurately identify various tiny lesion spots and small defects on apple surfaces, meeting the efficient operation requirements of large-scale orchard screening and industrial detection scenarios. 

Despite the promising performance demonstrated by the proposed model, several limitations should be acknowledged. First, the model may exhibit reduced detection accuracy under extreme lighting conditions, such as strong direct sunlight or heavy shadowing, which can significantly alter the visual appearance of disease symptoms. Second, although the model performs well on the four apple disease categories evaluated in this study, its generalization to other fruit types or different disease manifestations requires further validation. Third, the current approach relies on high-quality image acquisition; in scenarios involving motion blur, low-resolution captures, or severe occlusions by overlapping leaves and branches, detection performance may degrade.

For the follow-up research plan, this paper will expand the optimized YOLOv11s model to the surface defect detection tasks of various fruits, such as pears, peaches, and citrus, further optimize the model’s generalization ability and lightweight degree, strive to develop a universal and high-performance intelligent detection model for fruit surface defects, and promote the intelligent and large-scale development of fruit quality detection technology.

## Figures and Tables

**Table 1:** Main hyperparameters of training and preprocessing.

Training Hyperparameters	Value	Data Preprocess Hyperparameters	Value
Optimizer	Sgd	Mixup	0.0
Initial Learning Rate	0.001	Hsv_h	0.015
Final Learning Rate	0.00001	Hsv_s	0.7
Works	8	Hsv_v	0.4
Seed	0	Scale	0.5
Cos_lr	False	Flipud	0.0
Patience	100	Fliplr	0.5
Freeze	None	Translate	0.1
Mask_ratio	4	Copy_paste	0.0
Epochs	500	Erasing	0.4
Batch	100	Crop_fraction	1.0
Dropout	0.0	Copy_paste_mode	Flip

**Table 2:** Comparison results of dataset augmentation performance.

Datasets	Model	P (%)	R (%)	F1 (%)	mAP@50 (%)	mAP@50:95 (%)
Original dataset	Yolov10s	0.851	0.809	0.830	0.864	0.767
	Yolov11s	0.888	0.823	0.854	0.897	0.774
Augmented dataset	Yolov10s	0.897	0.851	0.873	0.912	0.805
	Yolov11s	0.929	0.847	0.886	0.926	0.813

**Table 3:** Comparison results of category-wise prediction values before and after dataset augmentation.

Datasets	Model	Healthy Apple	Blotch Apple	Rot Apple	Scab Apple	All Class mAP@0.5
Original dataset	Yolov10s	0.907	0.861	0.794	0.894	0.864
	Yolov11s	0.914	0.887	0.815	0.892	0.877
Augmented dataset	Yolov10s	0.933	0.942	0.856	0.917	0.912
	Yolov11s	0.939	0.950	0.888	0.927	0.926

**Table 4:** Ablation experiment results.

Model	P (%)	R (%)	F1 (%)	mAP@50 (%)	mAP@50:95 (%)	F1 Mean	F1 Variance
Model 1	0.929	0.847	0.886	0.926	0.813	0.8844	2.58 × 10^−6^
Model 2	0.924	0.866	0.894	0.930	0.818	0.8938	2.33 × 10^−6^
Model 3	0.933	0.881	0.906	0.936	0.825	0.9037	2.21 × 10^−6^
Model 4	0.942	0.893	0.917	0.939	0.831	0.9158	2.28 × 10^−6^
Model 5	0.947	0.910	0.928	0.943	0.837	0.9279	2.19 × 10^−6^

**Table 5:** PR values of five models for four detection targets.

Model	Healthy_apple	Blotch_apple	Rot_apple	Scab_apple	All Class mAP@0.5
Model 1	0.909	0.950	0.888	0.933	0.920
Model 2	0.911	0.965	0.889	0.947	0.928
Model 3	0.917	0.962	0.914	0.939	0.933
Model 4	0.922	0.974	0.918	0.946	0.940
Model 5	0.926	0.977	0.922	0.947	0.943

**Table 6:** Comparison results of different model performances.

Model	P (%)	R (%)	F1 (%)	mAP@50 (%)	mAP@50:95 (%)	FLOPs (G)	Params (M)	FPS
Yolov11s (improved)	0.947	0.910	0.928	0.943	0.837	22.9	10.6	551
Yolov11s	0.929	0.847	0.886	0.926	0.813	27.9	10.6	412
Yolov10s	0.897	0.851	0.873	0.912	0.805	26.8	9.4	577
Yolov9s	0.899	0.853	0.875	0.911	0.799	30.6	12.8	343
Yolov8s	0.897	0.848	0.871	0.895	0.787	28.6	11.2	461
Yolov6s	0.883	0.850	0.866	0.897	0.780	44.2	17.2	354
Faster R-CNN	0.902	0.857	0.879	0.884	0.783	22.7	31.5	54

## Data Availability

The raw data supporting the conclusions of this article will be made available by the authors on request.
